# 6,126 hepatectomies in 2022: current trend of outcome in Italy

**DOI:** 10.1007/s00423-024-03398-6

**Published:** 2024-07-10

**Authors:** Marco Nicolazzi, Marcello Di Martino, Paolo Baroffio, Matteo Donadon

**Affiliations:** 1 Department of Health Sciences, University of Piemonte Orientale, Novara, 28100 Italy; 2 Department of Surgery, University Maggiore Hospital della Carità, Corso Mazzini 18, Novara, 28100 Italy

**Keywords:** Liver resection, Liver surgery, Hepatectomy, Hepatocelullar carcinoma, Colorectal liver metastases, Cholangiocarcinoma

## Abstract

**Purpose:**

Whether hospital volume affects outcome of patients undergoing hepatobiliary surgery, and whether the centralization of such procedures is justified remains to be investigated. The aim of this study was to analyze the outcome of liver surgery in Italy in relationship of hospital volume.

**Methods:**

This is a nationwide retrospective observational study conducted on data collected by the National Italian Registry “Piano Nazionale Esiti” (PNE) 2023 that included all liver procedures performed in 2022. Outcome measure were case volume and 30-day mortality. Hospitals were classified as very high-volume (H-Vol), intermediate-volume (I-Vol), low-volume (L-Vol) and very low-volume (VL-VoL). A review on centralization process and outcome measures was added.

**Results:**

6,126 liver resections for liver tumors were performed in 327 hospitals in 2022. The 30-day mortality was 2.2%. There were 14 H-Vol, 19 I-Vol, 31 L-Vol and 263 VL-Vol hospitals with 30-day mortality of 1.7%, 2.2%, 2.6% and 3.6% respectively (*P* < 0.001); 220 centers (83%) performed less than 10 resections, and 78 (29%) centers only 1 resection in 2022. By considering the geographical macro-areas, the median count of liver resection performed in northern Italy exceeded those in central and southern Italy (57% vs. 23% vs. 20%, respectively).

**Conclusions:**

High-volume has been confirmed to be associated to better outcome after hepatobiliary surgical procedures. Further studies are required to detail the factors associated with mortality. The centralization process should be redesigned and oversight.

**Supplementary Information:**

The online version contains supplementary material available at 10.1007/s00423-024-03398-6.

## Introduction

The quality assessment of surgical procedures is becoming one of the priorities for different stakeholders involved in the healthcare system. Such assessment is crucial for any type of surgical procedures but for highly complex surgical procedures, such as hepatobiliary surgery, is even more crucial considering the associated morbidity and mortality risks [1–3]. As supported by a long-standing body of the literature, the centralization of complex surgery serves to increase the quality of care following the principle that more experience resulting from a larger number of patients treated is directly associated with better surgical outcome [4]. However, there is still no agreement among experts on precise criteria on what to centralize, where to centralize, and who should be entitled to perform complex procedures such as hepatobiliary surgery [5]. Besides, there is still no agreement on which outcome measures should be considered to assess quality and safety in surgery [6].

With the aim to refresh the discussion on quality assessment and centralization in liver surgery, here we sought to report the trend of outcome of liver surgery in Italy by using the annual cases volume collected by the *“Piano Nazionale Esiti”* (PNE) in 2023 [7], with the focus on the effect of hospital volume on surgical outcome. Moreover, we offered a narrative review on the centralization concept, and on different outcome measures that can be used as quality and safety metrics in liver surgery.

## Methods

### Study definition and source of data

This was a nationwide retrospective study of patients who underwent liver procedures for malignant liver tumors in Italy from January 1, 2022 to December 31, 2022. Data were obtained from the anonymized records of National Italian Registry PNE 2023, which is the last published by the Italian National Agency for Regional Health Services [7]. PNE collects information from all the Italian hospitals, both public and private, using the hospital discharge forms, based on the International Classification of Diseases, Ninth Revision (ICD-9-CM) [8]. The study was developed and presented according to Strengthening the Reporting of Observational Studies in Epidemiology [9].

### Variable of interest and outcomes

PNE data included the case volume of liver resection per hospital, both public and private, from January 2022 to December 2022 and the crude 30-day mortality rate, meaning the death during the hospitalization and/or within the first 30 days after operation for patients submitted to liver resection from January 2020 to December 2022. Included hospital were classified according to:

- The twenty Italian administrative regions [10].
- The three Italian macro-regions [10]: northern, central and southern Italian regions.
- The case volume during 2021 according to the definition proposed by the by Torzilli et al. [11] that identify three categories: (a) high volume (H-Vol), meaning more than 100 resections per year; (b) intermediate volume (I-Vol), meaning 51–100 resections per year and (c) low volume (L-Vol), meaning 21–50 resections per year. An additional category of hospital performing 20 or less liver resections, defined as very-low volume (VL-Vol) was added to the present analysis (10).

The primary endpoints of this study were:

- the case volume in the year 2022;
- the crude 30-day mortality rate in the available study period 2020–2022;
- the elaboration of a narrative review on centralization surgical volumes and outcome measures.

### Snapshot on cancer center centralization and outcome measures in liver surgery

A snapshot on cancer center centralization and outcome measures was prepared by using Medline to identify relevant articles published before the 31st of July 2023, using a combined text and MeSH search strategy. The search terms included centralization, volume, post-operative mortality, morbidity, benchmarking, textbook outcome, liver surgery, liver resection and hepatectomy. The search was limited to articles published in English in the last 10 years, and it was further broadened by extensive cross-checking of all the references in the articles retrieved to identify eventual additional non-indexed literature.

### Statistical analysis

Baseline characteristics of the study population were expressed as absolute numbers and relative frequencies measurements for qualitative variables, whereas continuous variables were presented as means with standard deviations (SD) if normally distributed, and non-normal variables were presented as medians with interquartile ranges (IQR). Statistical analysis was performed using the χ2 test for comparison of categorical variables and the Student’s t and Wilcoxon signed-rank tests for normally and non-normally distributed continuous variables, respectively. A *P* value < 0.05 (two-tailed) was considered statistically significant. All the statistical analyses were performed using Stata version 16.0 (StataCorp) [12].

## Results

### Snapshot from “Piano Nazionale Esiti (PNE) 2023”

**Fig. 1:**
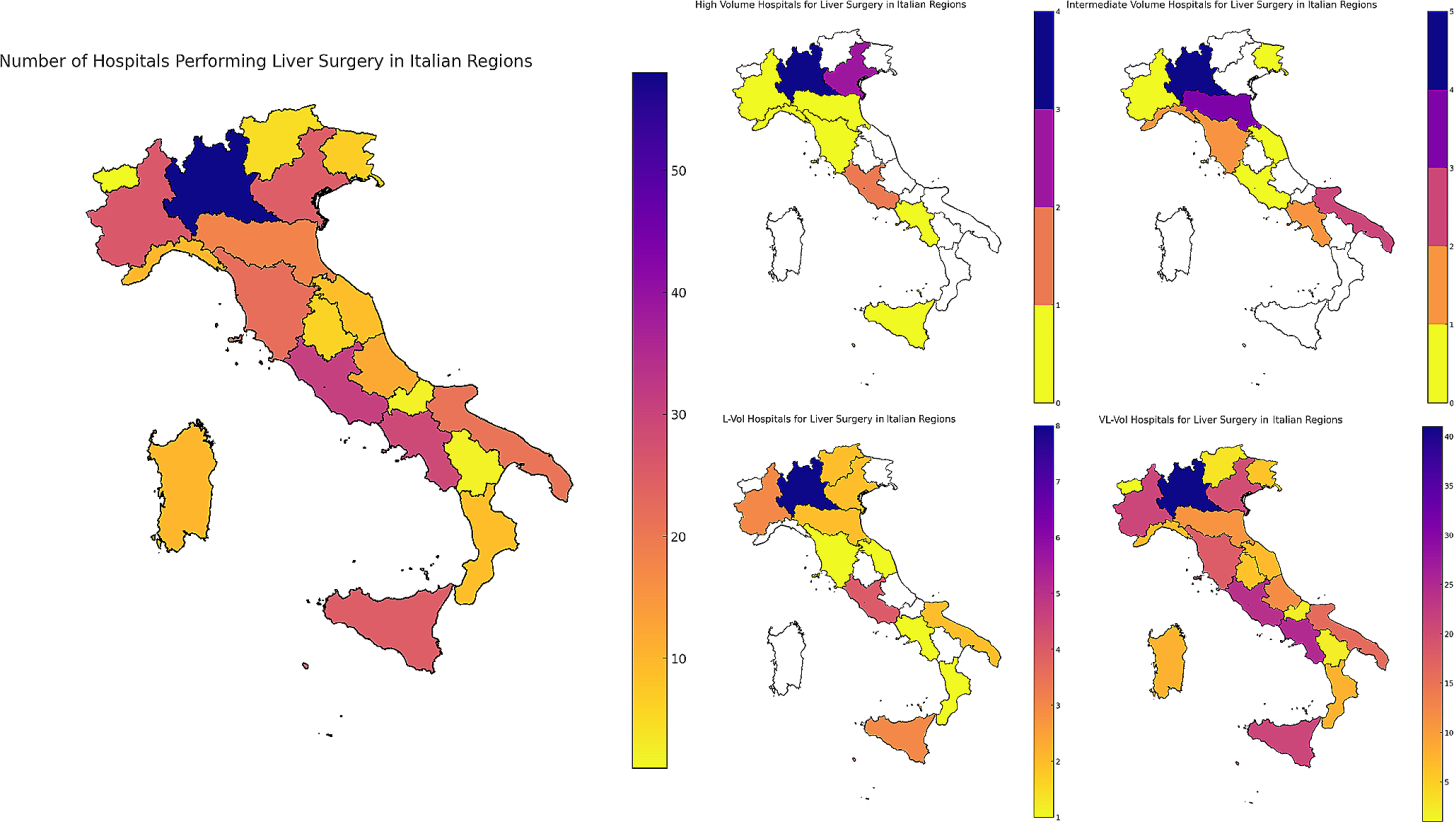
Map chart displaying the number of Italian hospitals performing liver surgery, along with the identification of regions categorized by high, intermediate, low, and very low surgical volume

**Fig. 2:**
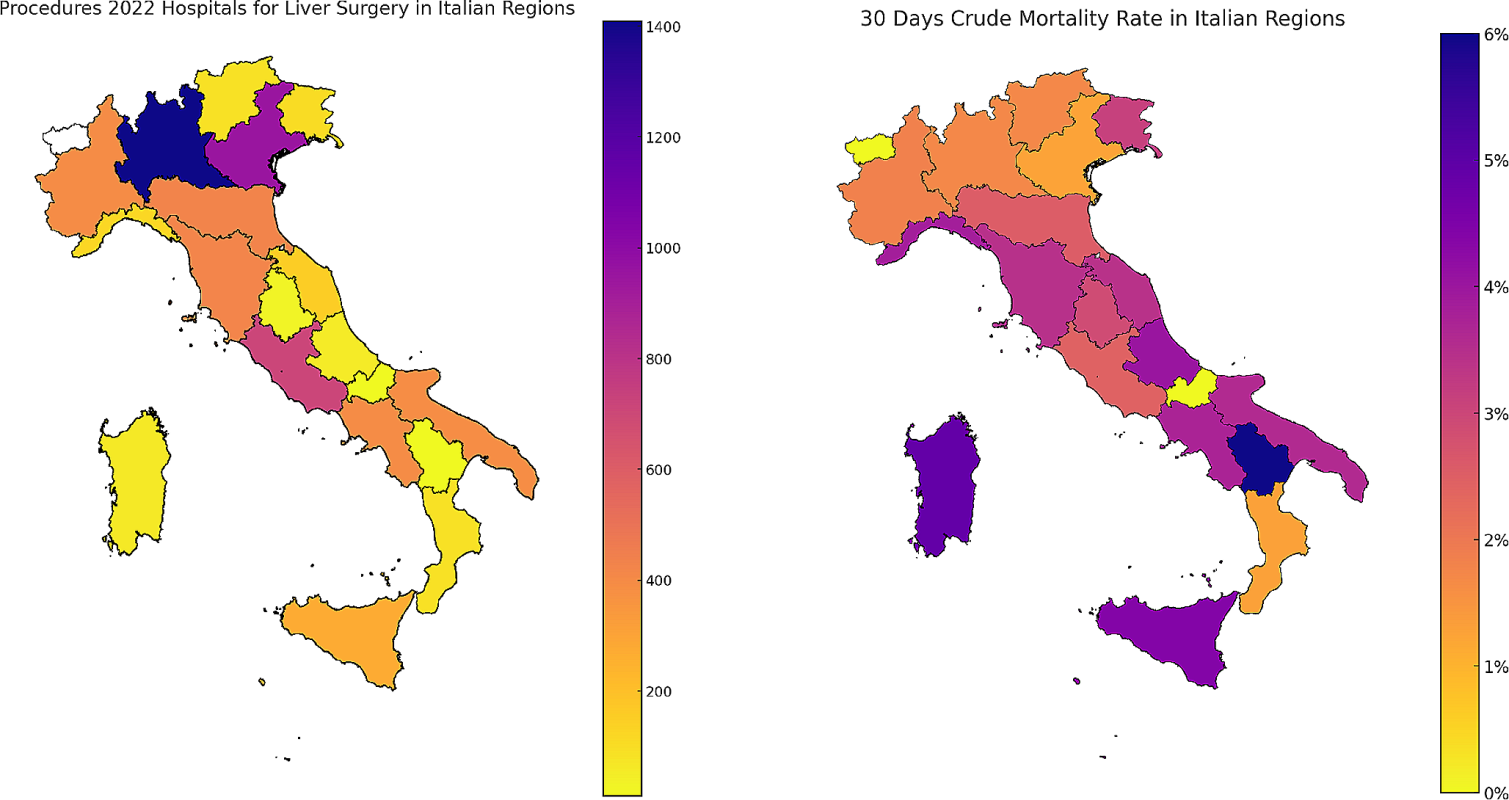
Map chart illustrating liver surgeries conducted across Italian regions in 2022, coupled with a representation of the 30-day crude mortality rate

In 2022, 6,126 liver resections for liver tumors were performed in 327 hospitals located in the 20 Italian administrative regions (Tables 1 and Fig. 1). There were 14 H-Vol centers, 19 I-Vol, 31 I-Vol and 263 VL-Vol centers. The total amount of procedures performed was 6,126 and the crude 30-day mortality rate for liver resection performed from 2020 to 2022 was 2.2% (Fig. 2).

**Table 1:** Snapshot from “Piano Nazionale Esiti (PNE) 2023”. Italian hospitals performing liver surgery according to the 20 administrative regions: volume, procedure performed and outcomes

	Number of hospitals	H-Vol	I-Vol	L-Vol		VL-Vol	Procedures 2022	Procedures 2020–2022	30 days crude mortality rate (2020–2022)
**Northern Italy**									
*Lombardia*	58	4	5	8	41		1409	2616	1.49%
*Piemonte*	26	1	1	3	21		395	820	1.59%
*Veneto*	25	3	-	2	20		951	1370	1.09%
*Emilia Romagna*	18	1	2	2	13		430	910	2.20%
*Liguria*	10	0	1	0	9		122	270	3.33%
*Trentino alto Adige*	5	-	-	2	3		91	202	1.48%
*Friuli Venezia Giulia*	7	-	1	1	5		106	260	2.69%
*Valle d’Aosta*	-	-	-	-	-		-	2	0%
**Central Italy**									
*Lazio*	31	2	1	4	24		714	1254	2.11%
*Toscana*	22	1	2	1	18		426	843	2.97%
*Marche*	9	-	1	1	7		158	270	2.96%
*Umbria*	6	-	.	-	6		32	120	2.50%
*Abruzzo*	12	-	-		12		56	144	3.48%
*Molise*	2	-	-	-	2		11	23	0%
**Southern Italy**									
*Campania*	29	1	2	1	25		395	840	3.22%
*Sicilia*	25	1	-	3	21		274	632	3.80%
*Calabria*	9	-	-	1	8		90	179	1.12%
*Sardegna*	10	-	-	-	10		62	165	4.24%
*Puglia*	21	-	3	2	16		390	771	3.11%
*Basilicata*	2	-	-		2		14	38	5.26%
**Total Italy**	327	14	19	31	263		6126	11,756	2.21%

H-Vol: high volume; I-Vol: intermediate volume; L-Vol: low volume; VL-Vol: very low volume

### Procedures performed and outcomes according to the three macro-areas

When considering the three Italian macro-areas, differences in the number of liver resections performed become evident. As outlined in Table 2, in the year 2022, 3,504 (57%) procedures took place in 150 (45.7%) hospitals in northern Italy, 1,397 (22.8%) in 82 (22.8%) hospitals in central Italy, and 1,225 (199.9%) in 96 (29.2%) hospitals in the southern Italy. Interestingly, the numbers of H-Vol and I-Vol hospitals were notably higher in the norther regions with 19 centers in the northern, 7 in the central and 7 in southern Italy. Besides, considering the inhabitants per macro-area (northern Italy ∼27 million; central Italy ∼13 million; southern Italy ∼18 millions) the distribution per inhabitant of H-Vol and I-Vol centers are significantly different: 1 center per 1,42 million of inhabitants in the north versus 1 center per 1,85 million of inhabitants in the center versus 1 center per 2,57 million of inhabitants in the south of Italy.

**Table 2:** Liver surgery according to the three Italian macro-areas: volume, procedure performed and outcomes

		Northern Italy	Central Italy	Southern Italy	*p*-value
Hospitals, n (%)		150 (45.7)	82 (25)	96 (29.2)	
Procedures performed during 2022, n (%)		3504 (57.1)	1397 (22.8)	1225 (19.9)	
Procedures performed by each hospital during 2022, median (IQR)		5 (2–20)	4 (2-11.5)	4 (1–11)	0.170
Hospitals by volume, n (%)	H-Vol	9 (6.4%)	3 (3.7%)	2 (2.0%)	0.471
	I-Vol	10 (6.7%)	4 (5.0%)	5 (5.1%)	
	L-Vol	18 (12.1%)	6 (7.5%)	7 (7.1%)	
	VL-Vol	112 (75.2%)	67 (83.7%)	84 (85.7%)	
30 days crude mortality rate from 2020–2022, n (%)		106 (1.6%)	68 (2.5%)	86 (3.2%)	< 0.001

IQR: interquartile range; H-Vol: high volume; I-Vol: intermediate volume; L-Vol: low volume; VL-Vol: very low volume

Notably, the hospital situated in the northern regions exhibited a significantly lower crude 30-day mortality rate in comparison with the central and southern regions (1.6% vs. 2.5% vs. 3.2%, respectively, *p* < 0.001).

### Procedures performed and outcomes according to hospital volume

A total of 14 (4.2%) centers were categorized as H-Vol, 19 (5.8%) as I-Vol, 31 (9.5%) as L-Vol, and 264 (80.4%) were classified as VL-Vol (Table 3). The distribution of liver resections performed in 2022 were as follows: 2,517 (41.1%) in H-Vol units; 1,292 (21.1%) in I-Vol units; 1,006 (16.4%) in L-Vol units and 1,311 (21.4%) in VL-Vol. Remarkably, still, in 2022, 2,317 (37.8%) procedures took place in L- and VL-Vol units. Notably, out of the 264 VL-Vol units, 220 (83%) carried out ≤ 10 resections, 183 (69%) performed ≤ 5 resections, and 78 (29%) conducted only 1 operation.

**Table 3:** Liver surgery according to hospital volume: procedures performed and outcomes

	H-Vol	I-Vol	L-Vol	VL-Vol	*p*-value
Hospitals, n (%)	14 (4.2)	19 (5.8)	31 (9.5)	263 (80.4)	
Procedures performed during 2022, n (%)	2517 (41.1)	1292 (21.1)	1006 (16.4)	1311 (21.4)	
Procedures performed by each hospital during 2022, median (IQR)	162 (123–208)	64 (60–80)	31 (23–40)	3 (1–7)	< 0.001
30 days crude mortality rate from 2019–2021, n (%)	110 (1.7%)	42 (2.2%)	52 (2.6%)	56 (3.6%)	< 0.001

IQR: interquartile range; H-Vol: high volume; I-Vol: intermediate volume; L-Vol: low volume; VL-Vol: very low volume

**Fig. 3:**
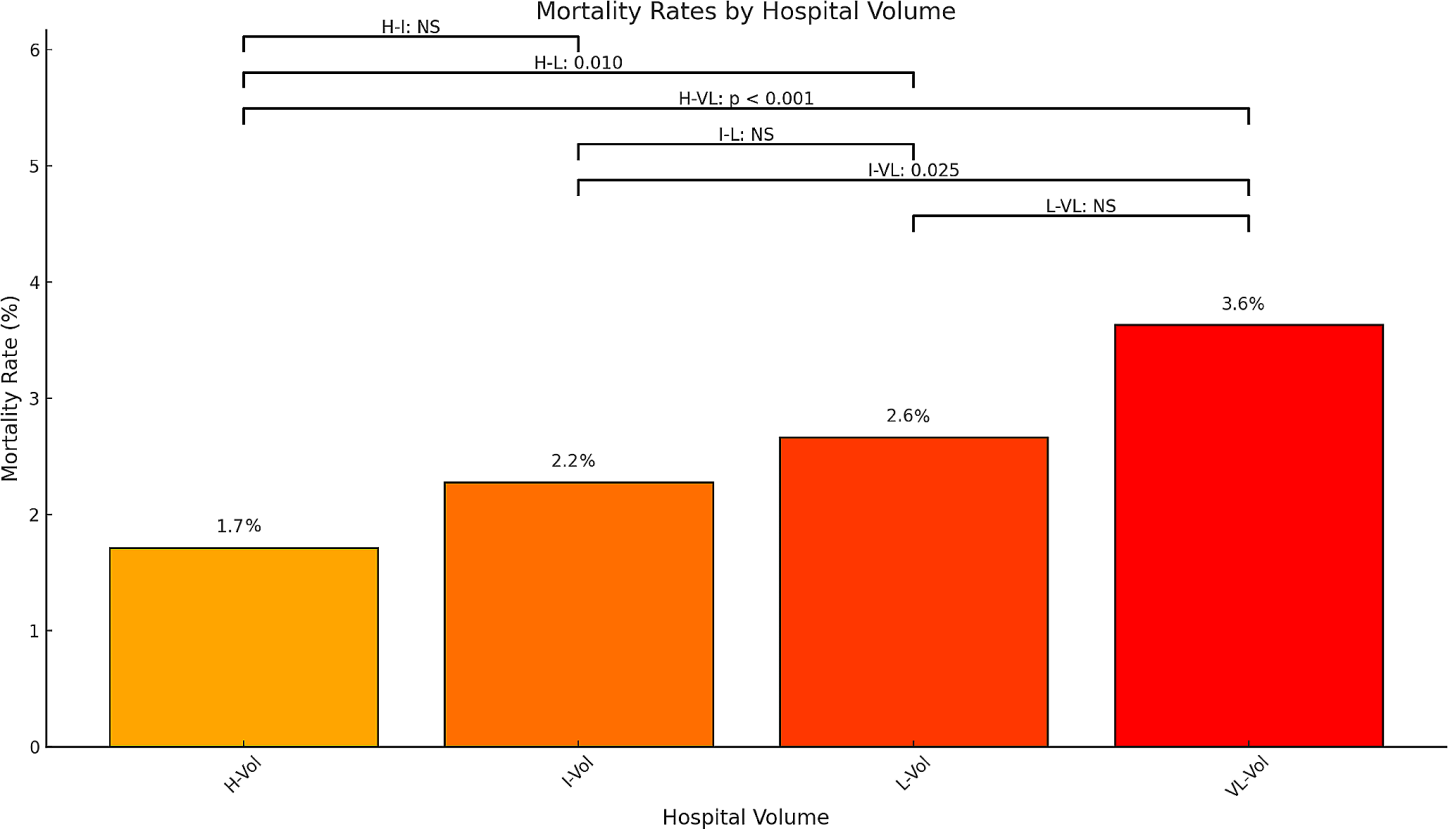
Mortality rate by volume of liver surgery in Italy during 2022

Interestingly, the median crude 30-day mortality rate stood at 1.7%, 2.2%, 2.6% and 3.6% for H-, I-, L- and VL-Vol centers, respectively (*p* < 0.001). Notably, VL-Vol hospitals exhibited an almost two-fold mortality rate compared to H-Vol centers (3.6% vs. 1.7%, p) (Fig. 3). This disparity was even more pronounced for centers that performed only 1 resection, where the mortality rate was 4.6%.

### Literature review on centralization, hospital volume and surgeon volume

#### Centralization

Centralization is defined as a process of concentration of resources, including staff, materials, infrastructures, knowledge, and expertise to enhance the quality of care achieving better outcomes. Consistently, centralization can be identified as the process that leads to the transfer from L-Vol centers to H-Vol centers, those complex and/or risky procedures that require more experiences, more resources (human, technical, structural) that are supposed to be insufficient in L-Vol centers.

Currently, in Italy the Regional Health Systems is based on the “hub-and-spoke” model [13]. Formally defined, the hub-and-spoke is a model that arranges service delivery assets into a network consisting of a leading center (hub) offering a full array of services, complemented by some secondary centers (spokes) that offer more limited services redirecting to the hub patients needing intensive care assistance [13]. Notably, this model is the one that is believed to be able to solve important disparities that limit patient access to specialized cures, such as those related to geographical territorial complexities, health professionals and technological resources which should contribute to limiting patient migration.

To date, there is a large gap between what is the hub-and-spoke model, as it is supposed to be, and what is in the real clinical practice: which cases or which pathologies are for hub and which are for spoke are, in general, not oversight by any local or central agencies. In 2018 a special committee from the European Surgical Association published a landmark paper [14] that listed twelve recommendations for the development of centralization strategies (Supplementary Table 1). To the best of our knowledge, these recommendations have not yet been fully considered nor applied by government agencies.

### Surgical volumes for liver surgery

#### Hospital volume

Considering that the centralization of major cancer surgery in hospitals with higher annual volume of procedures significantly reduces the risk of perioperative morbidity and mortality, as reported in the literature [11, 15–17], most of the efforts done so far were limited to listing the main hospital requirements. Based on the landmark paper by Torzilli et al. [11], three different types of requirements were identified for hepatobiliary surgery: hospital requirements, volume requirements, organization requirements:

##### Hospital requirements

According to experts and to the current national regulation “*Regolamento di definizione degli standard qualitativi, strutturali, tecnologici e quantitativi relativi all’assistenza ospedaliera” (Italian Law 135/2012)*, the hepatobiliary unit should be at least in a first level hospital that should have a series of units/services listed in Supplementary Table 2.

Moreover, H-Vol centers should either have a surgery unit dedicated to hepatobiliary surgery or a team of surgeons specialized in hepatobiliary surgery within a general/digestive surgery department.

##### Volume requirement

Every Hepatobiliary Unit, either as a Unit or as a team of dedicated surgeons inside a General/Digestive Surgery Division, should perform a minimum case volume per year. While no agreement exists on such a number, Torzilli et al. [11] in 2016 stated that at least 20 liver resections per year should be done for malignant diseases with 90-day mortality rate below 3%. Besides, three categories were identified according to the case volume: (i) L-Vol, meaning 21–50 resections per year; (ii) I-Vol, meaning 51–100 resections per year; (iii) H-Vol, meaning more than 100 resections per year. H-Vol centers should also be recognized as “referral units”, considering the high complexity of pathology, and as “units of excellence” when more than 5 scientific articles per year are published (6).

##### Organizations requirements

Hospitals should have Diagnostic-Therapeutic Flow Charts dedicated for patients with liver tumours. These charts should include any different type of liver disease and tumour, either primary or metastatic. Finally, importance for the multidisciplinary team assessment should be given, including physical space and quality working hours on a weekly basis.

The PNE data solely records hospital volume, without providing additional information on hospital and organisational requirements. This limitation precludes further interpretations.

#### Surgeon volume

Although hospital volume and surgeon volume are expected to correspond, this is not always the case in real-world practice. In hepatobiliary surgery, the relative importance of hospital volume versus surgeon volume is very important because both short- and long-term outcomes are dependent on hospital factors, such as the presence of an intensive care unit, and surgeon factors, such as operative technique. However, controversies exist among experts. Nathan et al. [3] showed that the protective effect of hospital hepatic resection volume persisted after case-mix adjustment for competing risk factors, while that was not the case considering the surgeon hepatic resection volume. Indeed, high- and low-volume surgeons had comparable in-hospital mortality rates after hepatectomy [3]. It is, anyway, a matter of fact that the experience of the surgeon represents a very important factor. Certainly, a better understanding of how the surgeon’s experience influences outcomes could help to develop specific healthcare strategies for improving the quality of care of hepatobiliary patients. One of the strategies could be the certification of the learning curve, that is the minimum number of procedures required to become fully proficient on a given specific hepatobiliary procedure. After that, the minimum case volume per year could become less important.

In relation to the PNE, it should be noted that there is no reporting of data on surgeons’ volume.

### Literature review on outcome measures: from the *crude mortality rate to composite outcomes for post-operative complications*

The mortality rate represents the number of deaths within a specific population during a defined time period. The crude mortality rate encompasses all causes of death within a given time interval for a particular population. On the other hand, the risk-adjusted mortality rate takes into account predictors of mortality, making it a more refined measure. Recently, with the enhancement of surgical safety and perioperative care, research emphasis has shifted from solely reducing mortality to also addressing postoperative complications and ensuring a satisfactory quality of life [18].

Postoperative complications signify deviations from the anticipated recovery trajectory following a surgical procedure. Various classification systems for postoperative complications have been proposed, as depicted in Table 4.

**Table 4:** Studies proving grading systems for post-operative complications

Study ID	Country	Title and acronym	Classification
Clavien 1992 (19)	Canada	Classification of surgical complications Also known as T92 score	4 categories
Pillai 1999 (21)	New Zealand	Complexity- and risk-adjusted model	12 variables
Dindo 2004 (20)	Switzerland	Classification of surgical complications. Also known as Clavien Dindo Classification (CDC)	5 categories
Martin 2022 (22)	USA	Memorial Sloan Kettering Cancer Center’s (MSKCC) Grading System.	5 categories
Strasberg 2009 (23)	Canada	Accordion Severity Grading System	6 categories
Slankamenac 2013 (24)	Switzerland	Comprehensive complication index (CCI)	Score from 0 to 100

Clavien and colleagues were the pioneers in proposing a classification for adverse post-operative outcomes, which garnered broad acceptance. In 1992 they introduced a standardized system or T29 score [19], later modified in 2004 by Dindo [20] resulting in the widely known and accepted Clavien and Dindo classification (CDC). Both systems were grounded in the patient’s health status and intervention requirements following surgery. While subsequent standards have emerged including the Complexity- and risk-adjusted model [21], the Memorial Sloan Kettering Cancer Center’s (MSKCC) grading system [22] and the Accordian severity system [23], the CDC remains the most prevalent method for grading post-operative complications. Nonetheless, a limitation of the CDC is its focus on the most severe complication experienced by the patient, disregarding less severe events and failing to capture the true overall complexity of adverse post-operative outcomes. To address this, the Comprehensive Complication Index (CCI) was developed in 2013 [24]. This index aggregates the complete burden of post-complications, considering their severity, into a unified score ranging from 0 to 100.

Furthermore, assessing the restoration of a good quality of life often employs quality of life questionnaires, such as the European Organisation For Research And Treatment Of Cancer (EORTC) Core Quality of Life questionnaire (QLQ-C30) [18]. These questionnaires are among the most widely accepted tools for evaluating quality of life.

To stay on PNE data, it records only 30-day mortality rates, which per se does not allow further interpretations. Moreover, 30-day mortality in liver surgery usually underestimates the mortality after hepatectomy, which should be recorded at 90-day [25].

### Benchmarking

In addition to the traditional outcome measures, hospitals and healthcare systems are progressively adopting composite tools designed to assess and enhance the quality of care. Benchmarking, characterized as a “continuous process of measuring products, services, and practices against the toughest competitors or those companies recognized as industry leaders” [26], serves as a quality improvement mechanism. It gauges the optimal attainable results within a group of well-defined, low-risk patients to establish meaningful reference values (benchmarks) for comparing outcomes [27]. The primary objective is to determine the most favourable achievable real-world postoperative outcomes [28]. Benchmarking has gained traction to evaluate and elevate the quality of care for patients undergoing liver resections [29–37] (Table 5).

**Table 5:** Studies on benchmarking in liver surgery

Study ID	Country	Benchmarks
Rossler 2016 (29)	Multicenter	Benchmarks for major liver surgery
Muller 2018 (30)	Multicenter	Benchmarks in liver transplantation
Bagante 2019 (31)	Multicenter	Benchmarks for complications after liver surgery
Russolillo (32)	Italy	Benchmarks in laparoscopic liver surgery
Famularo 2022 (33)	Italy	Benchmarks in open liver surgery for cirrhotic patients with hepatocellular carcinoma
Abbassi 2022 (35)	Multicenter	Benchmarks of redo liver transplantation
Goh 2023 (34)	Multicenter	Benchmarks in laparoscopic liver surgery
Fiorentini 2023 (36)	USA	Benchmarks of minimally invasive left lateral sectionectomy
Li 2023 (37)	Multicenter	Benchmark of adult-to-adult living-donor liver transplantation

The crucial steps in establishing a valid benchmark encompass: (a) selecting the intervention to be benchmarked; (b) identifying patient criteria that enable the selection of candidates; (c) defining specific key outcome indicators (benchmarks); (d) identifying eligible centres and patients; (e) calculating the benchmark values. One of the most widely accepted analytical strategies for defining benchmark values is the “Best Centre, Best Patients” (BCBP) approach. This involves selecting the best centre for treating a specific disease and focusing on low-risk patients. The benchmark values are then determined by calculating the 75th percentile for each centre based on specific outcomes. [29–31, 34]. An alternative analytical approach is the Achievable Benchmark of Care (ABC) method, which involves identifying the benchmark as the performance attained by the top 10% of providers, adjusted for the number of patients each provider treats [32, 33].

### Textbook outcomes

Another comprehensive multidimensional composite outcome indicator that encompasses the entirety of the surgical care process is the textbook outcome (TO). The TO strives to encapsulate the concept of an ideal “textbook” hospitalization, signifying patients who do not experience adverse outcomes following complex surgical procedures [38]. While the definition of textbook outcomes may differ based on the surgery type, it typically encompasses patients who do not encounter mortality, severe complications, readmission, and exhibit favorable surrogate oncological parameters. Numerous recent studies have focused on evaluating textbook outcomes in liver surgery [36, 38–42]. In 2021 Görgec et al. [39] published the findings of an international multicentric clinical study on textbook outcomes (TO) in liver surgery. The TO indicators encompassed the absence of: intraoperative events ≥ grade 2 based on the Oslo classification [43]; postoperative bile leak grade B or C according to the International Study Group of Liver Surgery classification [44]; severe complications ≥ grade III according to CDC [20], in-hospital mortality, postoperative reintervention, readmission, and the presence of R0 resection margin. A recent systematic review [42] provides an overview of the contemporary international experience with TO in evaluating surgical performance after liver surgery. The review suggests that TO serves as a unified composite metric that may offer a more patient-centered approach and is better suited for quantifying optimal care and facilitating performance comparisons among centers conducting liver surgery.

### Quality performance indicators

It’s important to note that while quality and safety are related, they are not synonymous. Safety pertains to preventing negative outcomes, whereas quality involves achieving positive outcomes. As previously mentioned, numerous factors contribute to safety, and in the recent times, the assessment of the quality of a specific surgical procedure is gaining increasing attention from international surgical associations. Woodhouse et al. [45] have recently formulated a set of globally accepted quality performance indicators (QPIs) for hepato-pancreato-biliary procedures. Through a modified Delphi process, three rounds of consultations were conducted with working groups comprising members of the International Hepato-Pancreato-Biliary Association (IHPBA). The final set of QPIs encompasses three categories: structure, process, and outcomes. A total of seven “core” indicators were unanimously agreed upon for liver, pancreatic, and complex biliary surgery, as outlined in Supplementary Table 3. Furthermore, an additional six procedure-specific QPIs were suggested for liver and complex biliary surgery, along with three for pancreatic surgery. These QPIs can be employed to measure and monitor the entire global process of liver surgery at an individual, unit, institutional, and/or jurisdictional level. They encompass not just clinical outcomes, but also structural and procedural characteristics. In this way, they encourage ongoing advancement and enhancement of safe and high-quality hepato-pancreato-biliary surgery on a global scale.

## Discussion

The increased demands for quality assessment in liver surgery together with the expected increasing incidence of primary liver tumors in the next few years [11, 45], justified the need to conduct research studies of this type.

The aim of this study was to provide a snapshot of the current trends of outcome of liver surgery in Italy using the data from PNE 202, which refers to the year 2022 that is the last available. By looking at those numbers, it is clear that the centralization of liver surgery in Italy is far from being operational: 14 H-Vol centers performed 41% of the liver resections while 263 VL-Vol centers perform 21% of those cases. Twenty different regional health systems under the National health system should provide more H-Vol centers, at least one per region, and certainly should work to limit the dispersion of a handful of cases in these VL-Vol centers. Based on that snapshot, it appears clear that the hub-and-spoke model does not work.

Consistently, the mortality rate in these VL-Vol centers was almost two-fold the mortality rate of the H-Vol centers (3.6% vs. 1.7%). Of note, 3.6% of 30-day mortality rate could apparently be considered adequate in liver surgery except that VL-Vol centers almost surely performed small, limited resections that, probably, could have been at lower morbidity and mortality risks if they would have been performed in H-Vol centers. In other words, it is likely that H-Vol performed complex cases while L- and VL-Vol centers did not. Besides, there is a trend of association of mortality that decreases by passing from very VL-Vol to L-Vol, then from I-Vol to H-Vol centers. These results are consistent with previous studies, indicating the relationship of hospital volume with postoperative mortality [5, 11, 17]. Unfortunately, PNE data do not include hospital and organisational data, patients’s characteristics, data on morbidity, type of complications details, failure to rescue and 90-day mortality rate that, if available, would allow more analyses. In particular, 90-day mortality should be used instead of 30-day mortality in hepatobiliary surgery to catch, for instance, those cases of post-hepatectomy liver failure that may become irreversible more than one month after the operation [46]. It is not surprising, however, that when 90-day mortality is available the association between hospital volume and outcome still remains significant. Guglielmi et al. [5], in fact, recently reported about the trends in hospital volume and mortality in hepatobiliary surgery in the Veneto region confirming an increased and significant risk of 30- and 90-day mortality in L-Vol centers.

While the metric of quality is a complex process of which a given threshold case volume is just a proxy measure, it is clear that the mortality risk after hepatectomy in Italy decreases when more than 20 resections per year are considered (3.6% vs. 2.6%). Indeed, Dimick et al. [47] already reported that those hospitals that performed more than 20 liver resections per year had significantly lower mortality rate (3.9% vs. 7.6%) even at L-Vol hospitals. The same result was reported for minimally invasive liver surgery by Van der Poel et al. [48], who showed that when more than 20 minimally invasive liver surgery per year are performed the risks of conversion and complication significantly decrease. Similarly, Ardito et al. [49] reported that failure to rescue, that is the mortality after postoperative complications, was lower in H-Vol centers compared to L-Vol centers indicating how the case volume is a measure of the experience of the surgeons team in identifying and treating complications that, if unrecognized, could lead to death.

What should not be underestimated are the social and economic implications of an unequal distribution of high-volume (H-Vol) and possibly more efficient healthcare centers across the Italian territory. In fact, a significant number of patients are willing to migrate to other regions in search of more efficient healthcare facilities where waiting times before an interventional procedure are usually shorter, and outcomes appear to be better. According to the 2020 AGENAS [12] report on healthcare mobility, healthcare expenditure is strongly impacted by healthcare migration, which continues to occur from southern to northern regions. In fact, 97% of the positive balance goes to the coffers of Lombardy, Emilia Romagna, Veneto, and Tuscany (with 697.6, 338.4, 142.9, and 125.6 million euros, respectively), while 76% of the negative balance is carried by Puglia, Sicily, Lazio, Calabria, and Campania (with 192.3, 212.8, 215.9, 280.5, 319.7 million euros, respectively). This aspect should not be underestimated when analyzing the global outcomes of a surgical procedure such as liver surgery at both the national and regional levels.

The centralization process of liver surgery in many countries including Italy should be redesigned. New ideas should be given. One could be to use a twin-track approach, which could save quality and safety for patients and, at the same time, it could preserve the health care professionals that work in L-Vol hospitals: complex cases, either for tumoral presentation, for patient’s status or for surgical approach required, should be centralized in H-Vol centers (hub) while standard cases could be decentralized in I- or even L-Vol centers (spoke) performing more than 20 resections per year. VL-Vol centers (1,351 resections in 2021) should be regulated. By using this twin-track approach more safety and quality in liver surgery should be warranted. Importantly, an efficient and oversight hub & spoke model would also warrant the education and training process of young surgeons, which should be rethought [50].

In the lack of standard outcome measures, which would allow more truly comparison among centers, we here proposed a narrative review on the current outcome metrics adopted in liver surgery aiming to convince the different involved stakeholders, including patients and regulatory agencies, that the metric of quality is a complex process in which the simplification does not pay off. Indeed, the assessment of surgeon’s competency for high-risk procedures should be based on composite metrics, among which certainly the case volume together with the hospital requirements play a role. At the same time, surgeon credentialing should reflect real-world practice data rather than arbitrary benchmarks [51, 52]. A more comprehensive quality measure would come by the diffusion of the new QPIs recently proposed by the IHPBA that being a summary of composite outcomes approved by an international committee should give back a more reliable quality tool [45]. What is still missing in all these types of outcome measures and quality metrics proposed, is the metric around the patient. The age, the performance status, and the presence of comorbidity cannot be neglected as factors associated with the outcome. The use of raw indices (morbidity and mortality rates) cannot allow a true comparison among hospitals, even though in large numbers one could argue that these patient’s factors might be considered well-distributed. However, in the absence of detailed data, this assumption remains a speculation.

This study has several limitations. First, the scarcity of available data from PNE does not allow further analyses, and interpretations. Factors affecting mortality are not reported and, as said, hospital volume acts just as a proxy measure of quality. Second, data from a single year (2022) may not be representative and may not be generalized as a global perspective of the Italian experience in liver surgery. However, this nationwide study on a very large population offers a snapshot of the current trends of outcome after hepatectomy in Italy that may serve as a basis for further considerations and improvements.

In conclusion, this study showed that the centralization process with the hub-and-spoke model for liver surgery in 2022 in Italy was mostly disregarded. Approximately 41% of resections were centralized in higher volume centers with expected decreased mortality. The threshold of 20 cases per year is confirmed to be the minimum case volume. Further studies are required to better detail the factors associated with failure to rescue, and mortality and then, to better detail what is complexity in liver surgery and which centers should be entitled and qualified to perform hepatobiliary surgery.

### Electronic supplementary material

Below is the link to the electronic supplementary material.

Supplementary Material 1

## Data Availability

The data that support this study are available from the corresponding author upon reasonable request.

## References

[1] Tol JA, van Gulik TM, Busch OR, Gouma DJ (2012). Centralization of highly complex low-volume procedures in upper gastrointestinal surgery. A summary of systematic reviews and meta-analyses. Dig Surg.

[2] Romatoski KS, Chung SH, de Geus SWL, Papageorge MV, Woods AP, Rasic G, Ng SC, Tseng JF, Sachs TE (2023). Combined high-volume common Complex Cancer Operations Safeguard Long-Term Survival in a low-volume Individual Cancer Operation setting. Ann Surg Oncol.

[3] Nathan H, Cameron JL, Choti MA, Schulick RD, Pawlik TM (2009). The volume-outcomes effect in hepato-pancreato-biliary surgery: hospital versus surgeon contributions and specificity of the relationship. J Am Coll Surg.

[4] Volume-Outcome (2021) Relationship in oncological surgery. Springer

[5] Guglielmi A, Tripepi M, Salmaso L, Fedeli U, Ruzzenente A, Saia M (2023) Trends in hospital volume and operative mortality in hepato-biliary surgery in Veneto region, Italy. Updates Surg 10.1007/s13304-023-01574-9 PMC10543584 37395932

[6] Woodhouse B, Panesar D, Koea J (2021). Quality performance indicators for hepato-pancreatico-biliary procedures: a systematic review. HPB (Oxford).

[7] Agenas Programma nazionale Esiti (2022) [Internet]. https://pne.agenas.it. Accessed 22 August 2023

[8] World Health O (1978) International classification of diseases: [9th] ninth revision, basic tabulation list with alphabetic index. In. Geneva: World Health Organization

[9] von Elm E, Altman DG, Egger M, Pocock SJ, Gotzsche PC, Vandenbroucke JP, Initiative S (2007). Strengthening the reporting of Observational studies in Epidemiology (STROBE) statement: guidelines for reporting observational studies. BMJ.

[10] Statistical regions in the European Union and partner countries NUTS and statistical regions 2021-re-edition (2022) [Internet]. https://ec.europa.eu/eurostat/web/products-manuals-and-guidelines/-/ks-gq-22-010. Accessed 22 August 2023

[11] Torzilli G, Vigano L, Giuliante F, Pinna AD (2016). Liver surgery in Italy. Criteria to identify the hospital units and the tertiary referral centers entitled to perform it. Updates Surg.

[12] Mobilità Sanitaria: le chiavi di lettura dell’ agenzia (2020) [Internet]. https://www.agenas.gov.it/images/agenas/In%20primo%20piano/2020/novembre/slide_mobilita.pdf. Accessed 12 September 2023

[13] Elrod JK, Fortenberry JL (2017). The hub-and-spoke organization design: an avenue for serving patients well. BMC Health Serv Res.

[14] Vonlanthen R, Lodge P, Barkun JS, Farges O, Rogiers X, Soreide K, Kehlet H, Reynolds JV, Kaser SA, Naredi P (2018). Toward a Consensus on centralization in surgery. Ann Surg.

[15] Busweiler LAD, Dikken JL, Henneman D, van Berge Henegouwen MI, Ho VKY, Tollenaar R, Wouters M, van Sandick JW (2017). The influence of a composite hospital volume on outcomes for gastric cancer surgery: a Dutch population-based study. J Surg Oncol.

[16] Chowdhury MM, Dagash H, Pierro A (2007). A systematic review of the impact of volume of surgery and specialization on patient outcome. Br J Surg.

[17] Gruen RL, Pitt V, Green S, Parkhill A, Campbell D, Jolley D (2009). The effect of provider case volume on cancer mortality: systematic review and meta-analysis. CA Cancer J Clin.

[18] Kaasa S, Bjordal K, Aaronson N, Moum T, Wist E, Hagen S, Kvikstad A (1995). The EORTC core quality of life questionnaire (QLQ-C30): validity and reliability when analysed with patients treated with palliative radiotherapy. Eur J Cancer.

[19] Clavien PA, Sanabria JR, Strasberg SM (1992). Proposed classification of complications of surgery with examples of utility in cholecystectomy. Surgery.

[20] Dindo D, Demartines N, Clavien PA (2004). Classification of surgical complications: a new proposal with evaluation in a cohort of 6336 patients and results of a survey. Ann Surg.

[21] Pillai SB, van Rij AM, Williams S, Thomson IA, Putterill MJ, Greig S (1999). Complexity- and risk-adjusted model for measuring surgical outcome. Br J Surg.

[22] Martin RC 2nd, Brennan MF, Jaques DP (2002) Quality of complication reporting in the surgical literature. Ann Surg 235(6):803–813 10.1097/00000658-200206000-00007 PMC1422509 12035036

[23] Strasberg SM, Linehan DC, Hawkins WG (2009). The accordion severity grading system of surgical complications. Ann Surg.

[24] Slankamenac K, Graf R, Barkun J, Puhan MA, Clavien PA (2013). The comprehensive complication index: a novel continuous scale to measure surgical morbidity. Ann Surg.

[25] Mayo SC, Shore AD, Nathan H, Edil BH, Hirose K, Anders RA, Wolfgang CL, Schulick RD, Choti MA, Pawlik TM (2011). Refining the definition of perioperative mortality following hepatectomy using death within 90 days as the standard criterion. HPB (Oxford).

[26] Willmington C, Belardi P, Murante AM, Vainieri M (2022). The contribution of benchmarking to quality improvement in healthcare. A systematic literature review. BMC Health Serv Res.

[27] Sanchez-Velazquez P, Muller X, Malleo G, Park JS, Hwang HK, Napoli N, Javed AA, Inoue Y, Beghdadi N, Kalisvaart M (2019). Benchmarks in pancreatic surgery: a Novel Tool for unbiased outcome comparisons. Ann Surg.

[28] Staiger RD, Schwandt H, Puhan MA, Clavien PA (2019). Improving surgical outcomes through benchmarking. Br J Surg.

[29] Rossler F, Sapisochin G, Song G, Lin YH, Simpson MA, Hasegawa K, Laurenzi A, Sanchez Cabus S, Nunez MI, Gatti A (2016). Defining benchmarks for major liver surgery: a multicenter analysis of 5202 living liver donors. Ann Surg.

[30] Muller X, Marcon F, Sapisochin G, Marquez M, Dondero F, Rayar M, Doyle MMB, Callans L, Li J, Nowak G (2018). Defining benchmarks in Liver Transplantation: a Multicenter Outcome Analysis determining best achievable results. Ann Surg.

[31] Bagante F, Ruzzenente A, Beal EW, Campagnaro T, Merath K, Conci S, Akgul O, Alexandrescu S, Marques HP, Lam V (2019). Complications after liver surgery: a benchmark analysis. HPB (Oxford).

[32] Russolillo N, Aldrighetti L, Cillo U, Guglielmi A, Ettorre GM, Giuliante F, Mazzaferro V, Dalla Valle R, De Carlis L, Jovine E (2020). Risk-adjusted benchmarks in laparoscopic liver surgery in a national cohort. Br J Surg.

[33] Famularo S, Russolillo N, Donadon M, Cipriani F, Ardito F, Perri P, Giani A, De Stefano F, Lai Q, Molfino S (2022). Benchmarking postoperative outcomes after open liver surgery for cirrhotic patients with hepatocellular carcinoma in a national cohort. HPB (Oxford).

[34] Goh BKP, Han HS, Chen KH, Chua DW, Chan CY, Cipriani F, Aghayan DL, Fretland AA, Sijberden J, D’Silva M (2023). Defining global benchmarks for laparoscopic liver resections: an International Multicenter Study. Ann Surg.

[35] Abbassi F, Gero D, Muller X, Bueno A, Figiel W, Robin F, Laroche S, Picard B, Shankar S, Ivanics T (2022). Novel benchmark values for Redo Liver transplantation: does the Outcome justify the effort?. Ann Surg.

[36] Fiorentini G, Essaji Y, Geller DA, Iannitti DA, Baker EH, Warner SG, Sucandy I, Serrano PE, Onkendi E, Helton WS (2023). Textbook outcomes and benchmarks of minimally invasive left lateral sectionectomy across North America. Surg Endosc.

[37] Li Z, Rammohan A, Gunasekaran V, Hong S, Chen CY, Kim J, Hervera Marquez KA, Hsu SC, Kirimker O, Akamatsu N (2023). Novel benchmark for adult-to-adult living-donor liver transplantation.

[38] Halpern SE, Moris D, Shaw BI, Kesseli SJ, Samoylova ML, Manook M, Schmitz R, Collins BH, Sanoff SL, Ravindra KV (2021). Definition and analysis of Textbook Outcome: a Novel Quality measure in kidney transplantation. World J Surg.

[39] Gorgec B, Benedetti Cacciaguerra A, Lanari J, Russolillo N, Cipriani F, Aghayan D, Zimmitti G, Efanov M, Alseidi A, Mocchegiani F (2021). Assessment of Textbook Outcome in Laparoscopic and Open Liver surgery. JAMA Surg.

[40] de Graaff MR, Elfrink AKE, Buis CI, Swijnenburg RJ, Erdmann JI, Kazemier G, Verhoef C, Mieog JSD, Derksen WJM, van den Boezem PB (2022). Defining Textbook Outcome in liver surgery and assessment of hospital variation: a nationwide population-based study. Eur J Surg Oncol.

[41] Liu ZP, Guo W, Yin DL, Chen WY, Wang JY, Li XL, Yue P, Yu C, Wu ZP, Ding R et al (2023) Textbook outcomes in liver surgery for gallbladder cancer patients treated with curative-intent resection: a multicenter observational study. Int J Surg 10.1097/JS9.0000000000000510 PMC10498895 37288584

[42] Sweigert PJ, Ramia JM, Villodre C, Carbonell-Morote S, De-la-Plaza R, Serradilla M, Pawlik TM (2023). Textbook outcomes in Liver surgery: a systematic review. J Gastrointest Surg.

[43] Kazaryan AM, Rosok BI, Edwin B (2013) Morbidity assessment in surgery: refinement proposal based on a concept of perioperative adverse events. ISRN Surg, 2013:625093 10.1155/2013/625093 PMC3671541 23762627

[44] Koch M, Garden OJ, Padbury R, Rahbari NN, Adam R, Capussotti L, Fan ST, Yokoyama Y, Crawford M, Makuuchi M (2011). Bile leakage after hepatobiliary and pancreatic surgery: a definition and grading of severity by the International Study Group of Liver surgery. Surgery.

[45] Woodhouse B, Barreto SG, Soreide K, Stavrou GA, Teh C, Pitt H, Di Martino M, Herman P, Lopez-Lopez V, Berrevoet F (2023). A core set of quality performance indicators for HPB procedures: a global consensus for hepatectomy, pancreatectomy, and complex biliary surgery. HPB (Oxford).

[46] Gani F, Azoulay D, Pawlik TM (2017). Evaluating trends in the volume-outcomes relationship following liver surgery: does Regionalization Benefit all patients the same?. J Gastrointest Surg.

[47] Dimick JB, Cowan JA, Knol JA, Upchurch GR (2003). Hepatic resection in the United States: indications, outcomes, and hospital procedural volumes from a nationally representative database. Arch Surg.

[48] van der Poel MJ, Fichtinger RS, Bemelmans M, Bosscha K, Braat AE, de Boer MT, Dejong CHC, Doornebosch PG, Draaisma WA, Gerhards MF (2019). Implementation and outcome of minor and major minimally invasive liver surgery in the Netherlands. HPB (Oxford).

[49] Ardito F, Famularo S, Aldrighetti L, Grazi GL, DallaValle R, Maestri M, Jovine E, Ruzzenente A, Baiocchi GL, Ercolani G (2020). The Impact of Hospital Volume on Failure to Rescue after liver resection for Hepatocellular Carcinoma: analysis from the HE.RC.O.LE.S. Italian Registry. Ann Surg.

[50] Donadon M, Montorsi M (2023). Volume-outcome in oncological surgery: reflections on education and training. Updates Surg.

[51] Stone DH, Upchurch GR, Scali ST (2021). Surgeon Credentialing should reflect real-world practice outcomes rather than arbitrary minimum-volume benchmarks. JAMA Surg.

[52] Needleman BJ, Brethauer SA, Pawlik TM (2020). Assessing a surgeon’s competency for high-risk procedures: should we be looking at the bigger picture?. JAMA Netw Open.

