# Supplementary material for: 6,126 hepatectomies in 2022: current trend of outcome in Italy
Source: Langenbecks Arch Surg. 2024 Jul 10;409(1):211. doi: 10.1007/s00423-024-03398-6 (PMC11236879; doi:10.1007/s00423-024-03398-6)
Supplement: Supplementary file 1 — Supplementary Material 1 [file 423_2024_3398_MOESM1_ESM.docx]

**Supplementary Table 1**: European Surgical Association (ESA) recommendations

| Twelve Recommendations for Centralization |
| --- |
| 1 Definition should be based on disease (e.g.: pancreatic cancer) or organ systems (e.g.: complex HPB diseases) rather than procedure (e.g.: esophagectomy for pancreatectomy). |
| 2 The planning is based on minimal numbers of cases per center and also well distributed among various regions, considering population and cultural specificities in a country. |
| 3 Planning should include at least 2 centers per country to secure choice and competition (except for small countries and very rare diseases). |
| 4 Appropriate resources must be secured with proper evaluation, available infrastructure and personnel. |
| 5 Centers must offer fully functioning multidisciplinary teams (MDTs) of specialists capable of tackling all aspects of the diseases all year around. |
| 6 Centers must be linked to a network of hospitals to secure adequate referral and follow-up. |
| 7 Specifications of centralization must be legally enforced for adherence to specifications applied at the local and regional level and for private and non-private hospitals. |
| 8 The process for centralization must be accompanied by mainstream media activities to secure appropriate awareness of the population. |
| 9 Centers must have an externally audited database and are actively involved in clinical studies (including RCTs) and should be encouraged to contribute to laboratory research along with basic scientists. |
| 10 Quality control must be accompanied by international benchmark comparative studies. |
| 11 Equal accessibility to centralized healthcare should be monitored. |
| 12 Centers must be involved in surgical education, and secure specialized training as well as allowing rotation of ‘‘general surgeons’.’ |

**Supplementary Table 2**: Hospital requirements for liver Units as recommended by Italian Law 135/2012

| Hospital requirements |
| --- |
| Medical oncology |
| Hepatology or a team dedicated to the hepatology in an internal medicine unit |
| Radiology with ultrasonography, computed tomography, and magnetic resonance imaging |
| Interventional radiology (on call 24/7) |
| Digestive endoscopy, both diagnostic and interventional |
| Intensive care unit |
| Pathology, with the possibility to perform intraoperative frozen section examinations |
| Liver transplantation center (in the hospital or partnership) |
| Nuclear Medicine and Radiation Oncology (in the hospital or partnership) |

**Supplementary Table 3**: International Hepato-Pancreato-Biliary Association (IHPBA) Quality Performance Indicators (QPI) for liver surgery.

| Type | Quality Performance Indicators |
| --- | --- |
| Structure | Availability of specific services on site (including interventional radiology, endoscopy, intensive care unit, full range of diagnostic imaging, medical oncology, radiation oncology, hepatology (only required for liver resection) and at least two trained specialist surgeons (certified / fellowship training post-residency)* |
|  | Institution annual case load* |
|  | Specialized HPB multidisciplinary team (MDT) review - MDT containing trained surgeon, radiologist (interventional), medical oncologist, radiation oncologist and hepatologist/gastroenterologist* |
| Process | Synoptic pathology reporting (closest margin in mm, lymph node ratio). Note for liver resection: using specific templates for HCC, CholangioCA and CRLM report on liver (fibrosis, post-chemo injury etc)* |
|  | Synoptic operation report, including operative approach (open, lap, robotic), conversion rate (unplanned), operative time, blood loss, total Pringle time |
|  | Intra-operative transfusion |
|  | Pre-operative liver function - formal assessment (MELD, Child-Pugh) and calculated FLR |
|  | Post-hepatectomy liver failure - Grade B and above |
|  | R0/R1 rate |
| Outcome | Unplanned reintervention within 90-days (reoperation, interventional radiology, endoscopy* |
|  | Morbidity (Biliary leak rate & Major complications (Clavien-Dindo classification ≥III))* |
|  | 90-day mortality* |
|  | Readmission 30-day after discharge to any hospital |

*Core indicators
